# Direct Costs Vary by Outcome in Two-Stage Revision Arthroplasty for the Treatment of Hip Periprosthetic Joint Infection

**DOI:** 10.1016/j.artd.2022.10.011

**Published:** 2022-11-28

**Authors:** Colleen M. Wixted, Lefko T. Charalambous, Billy I. Kim, Niall H. Cochrane, Elshaday S. Belay, Hayden L. Joseph, Thorsten M. Seyler

**Affiliations:** a Duke University School of Medicine, Durham, NC, USA; b Duke University, Department of Orthopaedics, Durham, NC, USA

**Keywords:** Total hip arthroplasty, Prosthetic joint infection, Two-stage exchange arthroplasty, Surgical complications, Economic analysis, Cost analysis

## Abstract

**Background:**

Two-stage revision arthroplasty is the gold standard for treating chronic prosthetic joint infection (PJI), but there has been limited analysis of the costs incurred beyond the index procedure and how additional complications and/or surgeries impact the cost of care.

**Methods:**

The electronic health record was queried for patients who underwent a total hip arthroplasty complicated by PJI and then underwent removal of the prosthesis with implantation of an antibiotic-impregnated articulating cement spacer. Patient demographics, surgical variables, hospital readmissions, emergency department (ED) visits, and postoperative complications were recorded. Data on total costs were collected with an internal accounting database. The average follow-up duration was 3.35 years.

**Results:**

Univariate analyses showed statistically significant differences between outcome groups (reimplantation, reimplantation requiring later revision, retained spacer, and Girdlestone resection arthroplasty) in total overall costs, ED visit costs, and postoperative costs at 1 and 2 years after the initial spacer placement. The median total cost at 2 years for each group was $38,865 ($29,144-49,471) (reimplantation), $79,223 ($53,442-100,152) (reimplantation with revision), $54,096 ($20,872-73,903) (retained spacer), $62,134 ($52,135-101,546) (Girdlestone). Patients who underwent successful reimplantation requiring no further surgery had significantly lower total costs than patients who needed revision surgeries after reimplantation ($38,865 [$29,144-49,471] vs $79,223 [$53,442-100,152], *P* = .007). Patients with a Girdlestone resection arthroplasty had higher total costs at 1 year ($59,708 [$41,781-80,916] vs $33,093 [$27,237-40,429], *P* = .043) and higher costs attributable to ED visits at 2 years than the reimplantation group ($23,581 [$14,029-41,519] vs $15,307 [$6291-29,119], *P* = .009).

**Conclusions:**

A significant variation exists among total costs for the 2-stage treatment of hip PJI when stratified by the final outcome.

## Introduction

Prosthetic joint infection (PJI) is a devastating complication following primary total joint arthroplasty (TJA). The incidence of PJI has been estimated to range from 1% to 3% [[1], [2], [3], [4]] and accounts for 15% and 25% of revision total hip arthroplasty (THA) and revision total knee arthroplasty procedures, respectively [1,3]. The number of both primary and revision TJA procedures performed is expected to rise exponentially, and as a result, the incidence of PJI will continue to grow [5].

Treatment algorithms for PJI typically depend on the timing of symptom onset and the mechanism of infection. Acute PJI is often treated with debridement, antibiotics, and implant retention while patients with chronic PJI undergo 2-stage revision arthroplasty, the current gold-standard in North America [6]. Success rates of 2-stage revision procedures range from 70% to 90% [[7], [8], [9], [10], [11], [12]]. Although long-term infection control is attainable with these treatment strategies, there is substantial morbidity and mortality associated with them. Management of PJI often results in multiple inpatient hospitalizations and unplanned emergency department visits and readmissions and overall can negatively impact the patient’s quality of life and postoperative joint function [[13], [14], [15], [16]]. If the infection is not cleared after the reimplantation stage, patients may undergo additional debridement procedures, spacer exchanges, prolonged antibiotics, infection-related revision surgeries, or resection arthroplasty [17].

PJI significantly impacts not only the affected individual but also the broader health care system, especially as the push to control health care spending and shift to value-based reimbursement models accelerates. The economic burden associated with PJI was estimated to be around $1B in 2017 and is projected to reach $1.85B by 2030 [3,4]. With increases in TJA procedure volume acting as one of the primary drivers of these increases in cost, it is critical to understand the expense to treat a single PJI episode. Thus far, the total direct costs for the 2-stage treatment of PJI have been estimated to range from $30,000 to $100,000 [4,16,[18], [19], [20], [21]]. However, these studies involve heterogenous cohorts, may be performed in other countries, and most importantly, focus on costs associated with the index procedure, rather than on longitudinal costs that accumulate in the follow-up period. Because patients may experience interstage complications or fail to complete both stages of treatment in the case of 2-stage revisions, it is important to understand how variation in a patient’s final outcome can affect the total cost of care.

The aim of this study is (1) to quantify direct costs of 2-stage exchange arthroplasty with the placement of an articulating spacer for hip PJI in the 90-day, 1-year, and 2-year period; costs attributable to ED visits; and costs related to postoperative care and (2) to analyze the costs of PJI treatment stratified by the final outcome (reimplantation, reimplantation with revision, spacer, and Girdlestone/resection arthroplasty).

## Material and methods

### Patient selection

After receiving institutional review board approval, an institutional electronic health record database was retrospectively queried from January 2009 through December 2020 for patients who developed a PJI after THA with subsequent removal of the hip prosthesis and placement of an antibiotic-impregnated articulating cement spacer. All procedures were performed by 1 of 7 fellowship-trained surgeons at an academic tertiary referral center. Data were gathered through retrospective chart reviews and algorithmic queries of the institutional electronic health record database.

Patients aged 18 years or older with a diagnosis of PJI per the Musculoskeletal Infection Society criteria (score >6) who were subsequently treated at any time with an articulating spacer were included in the study [22]. Patients were excluded if they had less than 1 year of orthopedic follow-up, except patients who underwent reimplantation and were deemed infection-free by a team of infectious disease specialists. Patients who continued their care outside of the investigating institution’s primary health system were excluded as their cost data were unavailable for analysis. The mean follow-up duration for this cohort was 3.35 years (range 1.0-6.9 years).

### Data variables

Demographics of the patients were collected, including age, gender, race, ethnicity, and body mass index. Substance-use history (tobacco, alcohol, illicit drug use) and Elixhauser Comorbidity Index score were also collected using their respective International Classification of Diseases (ICD)-10 codes.

Data on hospital readmissions, emergency department (ED) visits, and complications following the initial surgery were collected. The primary outcome of this study was to determine the direct costs of 2-stage exchange arthroplasty with the placement of an articulating spacer for hip PJI in the 90-day, 1-year, and 2-year periods; costs attributable to ED visits; and costs related to postoperative care. These costs were then stratified by the patient’s final outcome (reimplantation, reimplantation with revision, spacer, Girdlestone/resection arthroplasty). Reimplantation was defined as reimplantation after spacer placement with no additional septic revision procedures. Reimplantation with revision was defined as reimplantation after spacer placement with additional septic revision procedures. Spacer was defined as the failure to complete the second-stage reimplantation at the time of final follow-up. Girdlestone/resection arthroplasty was defined as spacer explantation and subsequent removal of a portion of the hip joint.

Total costs from the initial surgery, additional subsequent surgeries, hospital readmissions, and ED visits through 2 years were captured through an internal accounting database (EPSi). EPSi prospectively tracks expenses at various phases of care throughout the hospital encounter (surgical, radiology, respiratory, pharmacy, intensive care services, cardiology, physical and occupational therapy, laboratory, transfusion, medical surgical supplies, and other direct costs). The time of encounter was defined as the time a patient was admitted to the hospital until the time they were discharged. All costs accrued from the initial surgical encounter through 2 years postoperatively were recorded. Costs for each patient were aggregated according to the service group. These were broken down as follows:

A. Intermediate service (nursing)
B. Intensive service (nursing)
C. Pharmacy service (all pharmaceuticals)
D. Surgery services (all surgical appliances and operating room–related charges)
E. Physical therapist, occupational therapist, and speech services
F. Radiology services
G. Labs (testing)
H. Emergency Department (ED) transport
I. Outpatient clinic (orthopaedic, infectious disease, plastic surgery follow-up care)

Costs not related to orthopedic care were excluded.

### Statistical analysis

Descriptive statistics of the cohort included both continuous and categorical variables that were represented as means or medians with standard deviations or interquartile ranges (IQRs) and counts and percentages, respectively.

Univariate analyses were performed to compare patient demographics and comorbidity variables across the final outcome groups. Costs were compared using nonparametric Mann-Whitney U tests. Other continuous or categorical variables were compared using t-tests, chi-square test, or Fisher’s exact test as appropriate. Post-hoc pairwise comparisons were performed between each final-outcome group to compare costs at each time point (total costs, initial spacer, ED visits, total postoperative cost). *P* values were corrected for multiple comparisons using the Bonferroni method.

## Results

### Patient demographics

In total, 102 hip PJIs were identified, with 47 excluded due to incomplete follow-up or cost data. A total of 55 hip PJIs were included in the final cohort. The mean age of the overall cohort was 62.5 years (SD: 11.4), and 54.5% of the cohort were female, with 85% reporting their race as Caucasian/White, 5.5% as African American, and 9.1% as other/unknown. The mean Elixhauser Comorbidity score was 6.78 (SD: 3.62), and the mean Elixhauser score of the Girdlestone/resection arthroplasty group was significantly higher than that of the other final outcome groups (mean: 11.00, *P* = .003). The remainder of patient demographics are recorded in Table 1.

**Table 1:** Patient demographics.

Demographic variable	Total cohort	Reimplantation	Reimplantation w/revision	Spacer	Girdlestone/resection arthroplasty	*P* value
	N = 55	N = 27	N = 11	N = 10	N = 7	
Age, mean (SD)	62.48 (11.40)	63.60 (9.10)	57.83 (9.98)	60.83 (15.71)	67.80 (13.85)	.286
Female sex, N (%)	30 (54.5)	15 (55.6)	6 (54.5)	6 (60.0)	3 (42.9)	.955
Race, N (%)						.364
Caucasian/White	47 (85.5)	24 (88.9)	9 (81.8)	7 (70.0)	7 (100.0)	
African American/Black	3 (5.5)	2 (7.4)	0 (0.0)	1 (10.0)	0 (0.0)	
Other/unknown	5 (9.1)	1 (3.7)	2 (18.2)	2 (20.0)	0 (0.0)	
Substance use history, N (%)						
Tobacco use	41 (74.5)	18 (66.7)	10 (90.9)	7 (70.0)	6 (85.7)	.391
Alcohol use	22 (40.0)	12 (44.4)	5 (45.5)	4 (40.0)	1 (14.3)	.552
Illicit drug use	5 (9.1)	1 (3.7)	2 (18.2)	1 (10.0)	1 (14.3)	.305
Elixhauser score, mean (SD)	6.78 (3.62)	5.70 (2.78)	7.45 (3.78)	6.00 (4.47)	11.00 (1.73)	.003^a^
Death, N (%)	3 (5.5)	0 (0.0)	1 (9.1)	1 (10.0)	1 (14.3)	.146
Follow-up time, d, median [IQR]	1154.00 [537.50, 1867.00]	1449.00 [889.50, 2052.50]	1154.00 [533.50, 1508.00]	792.50 [512.75, 1088.00]	1076.00 [692.00, 1823.50]	.110

a *P* value < .05.

### Total cost breakdown

**Figure 1:**
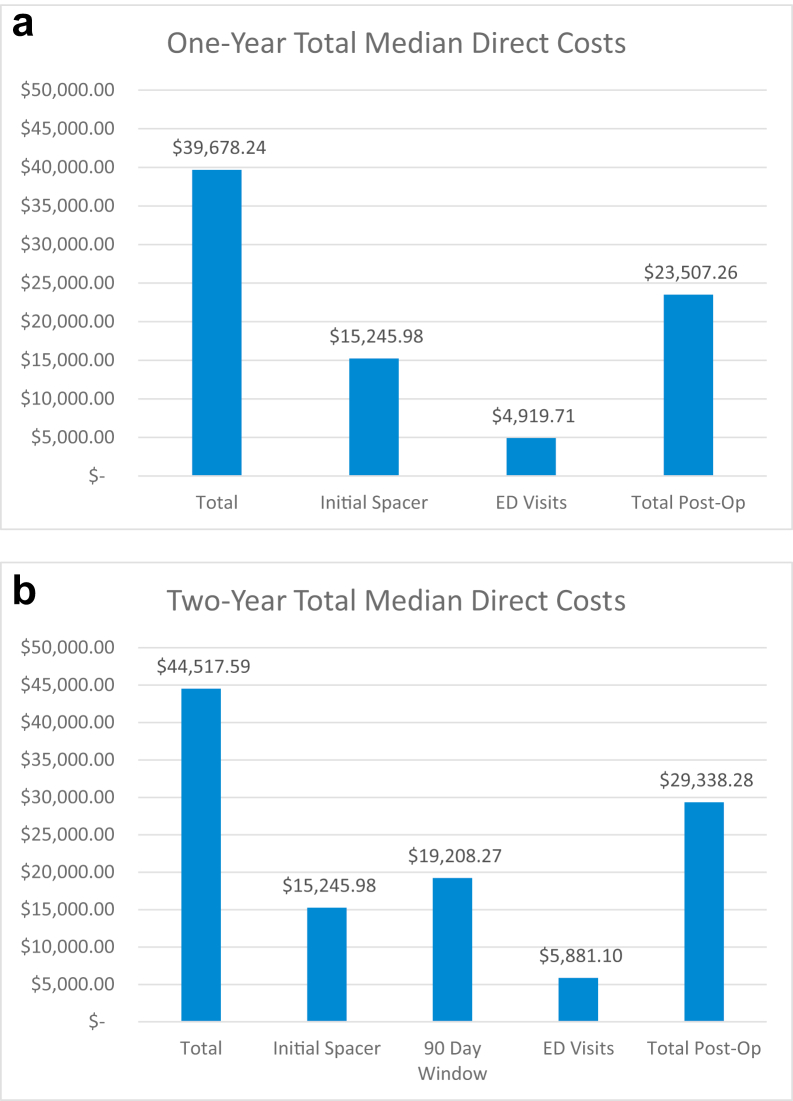
Charts of total median direct costs. (a) One year. (b) Two years.

Total costs at 1 and 2 years with breakdown by 90-day costs, costs at initial spacer placement, ED visits, and other postoperative costs are reported in Figure 1. Overall median total costs were $39,678 (IQR: $28,647-$64,947) at 1 year and $44,518 (IQR: $36,408-$76,091) at 2 years, with costs from initial spacer placement contributing a median of $15,245 (IQR: $13,328-$18,486). The median 90-day costs were $19,208 (IQR: $14,819-$30,894). Median costs attributable to ED visits were $4920 (IQR: $0-$22,927) at 1 year and $5881 (IQR: $0-$23,039) at 2 years. Other postoperative care costs included a median $23,507 (IQR: $12,782-$44,101) at 1 year and $29,338 (IQR: $20,135-$54,124) at 2 years. A detailed breakdown by cost service group for total overall costs is available in Table 2.

**Table 2:** Detailed breakdown of costs.

Service group	Year 1	Year 2
	Median cost ($USD) IQR	Median cost ($USD) IQR
Intermediate services	$6446.94 [4128.44, 9885.72]	$1742.57 [0.00, 7964.36]
Intensive nursing services	$0.00 [0.00,0.00]	$0.00 [0.00, 0.00]
Pharmacy services	$2127.90 [1351.52, 4093.88]	$856.29 [0.00, 3340.24]
Surgery services	$19,547.77 [12096.70, 24398.40]	$8776.89 [0.00, 19904.64]
PT/OT speech services	$893.13 [643.05, 1283.76]	$327.08 [0.00, 1066.11]
Radiology services	$373.45 [161.15, 684.52]	$211.56 [10.75, 574.24]
Labs	$1585.44 [1091.48, 2327.58]	$762.62 [74.78, 1566.40]
ER transport	$181.26 [0.00, 752.23]	$0.00 [0.00, 469.16]
Outpatient clinic	$106.87 [41.02, 186.25]	$69.05 [0.00, 215.75]

OT, occupational therapist; PT, physical therapist.

The majority of costs are contributed by surgical services during the first year (median: $19,548 [IQR: $12,097-$24,398]) and second year (median: $8,777 [IQR: $0-$19,905]) postoperatively. This is followed by nursing services (median: $6,447 [IQR: $4128-$9886]) and pharmacy services (median: $2,128 [IQR: $1352-$4094]) for the first year postoperatively. For the second year postoperatively, the next largest contributors to cost after surgical services are again nursing services (median: $1,743 [IQR: $0-$7964]) and pharmacy services (median: $856 [IQR: $0-$3340]).

### Costs stratified by final outcome

Costs stratified by the final outcome at 1 and 2 years after the initial spacer placement are provided in Table 3. There were statistically significant differences at all time points, except for initial spacer costs, between each final-outcome group. A further analysis revealed that patients who underwent 2-stage revision arthroplasty with successful reimplantation requiring no further surgery had total costs that were less than half of the costs for patients who did need revision surgeries after the second-stage reimplantation ($38,865 [$29,144-49,471] vs $79,223 [$53,442-100,152], *P* = .007). In addition, the total costs of postoperative care for patients who underwent reimplantation with more than 1 revision surgery were significantly higher at both 1 and 2 years (*P* = .0006 and *P* = .007, respectively). Patients whose final outcome was a Girdlestone resection arthroplasty had higher total costs at 1 year ($59,708 [$41,781-80,916]) than the reimplantation group ($33,093 [$27,237-40,429], *P* = .043). These patients also had higher costs attributable to ED visits through the 2 years of follow-up than the reimplantation group ($23,581 [$14,029-41,519] vs $15,307 [$6291-29,119], *P* = .009).

**Table 3:** Costs stratified by final outcome.

Cost window	Reimplantation (N = 27)	Reimplantation w/revision (N = 11)	Spacer (N = 10)	Girdlestone (N = 7)	*P* value
Costs at 1 y					
Total	$33,092.69 [272,37.03-404,29.04]	$79,223.43 [51,881.05-85,001.25]	$39,539.97 [18,271.22-62,761.37]	$59,708.45 [41,781.00-80,916.06]	.002^a^
90-d Window	$16,761.51 [145,91.10-224,45.73]	$30,923.79 [22,856.19-40,572.16]	$15,489.27 [14,736.64-24,207.38]	$28,384.95 [23,476.88-40,787.46]	.034^a^
Initial spacer	$15,225.67 [134,33.01-166,63.83]	$18,112.63 [14,623.08-22,714.34]	$15,039.25 [13,461.07-20,363.34]	$14,866.91 [13,596.80-20,574.43]	.591
ED visits	$0.00 [0.00-12,496.07]	$15,307.28 [5894.27-28,295.04]	$12,584.07 [29.34-15,393.81]	$23,039.44 [4969.46-34,763.73]	.037^a^
Total postoperative	$19,722.00 [12,370.93-24,607.35]	$45,865.45 [33,304.37-63,954.40]	$26,375.92 [1423.05-46,551.17]	$35,360.91 [24,179.63-40,243.49]	.004^a^
Costs at 2 y					
Total	$38,864.95 [29,144.33-49,471.08]	$79,223.43 [53,441.66-100,152.26]	$54,095.85 [20,871.69-73,902.64]	$62,134.40 [52,135.35-101,545.92]	.007^a^
ED visits	$0.00 [0.00-12,496.07]	$15,307.28 [6291.47-29,118.99]	$13,854.00 [1558.31-15,712.03]	$23,581.05 [14,029.32-41,519.35]	.005^a^

a *P* value < .05.

## Discussion

As reimbursement models continue to evolve and more focus is placed on quality of care and patient outcomes, health-care resource utilization will remain under scrutiny. In orthopaedics, bundled payments have become a common alternative payment model for various procedures, including TJA. While this incentivizes both increased cost-savings and high-quality care, providers and payors must be able to accurately estimate the cost of an episode of care to ensure sustainability of the model. Our study focused on the treatment of PJI after THA and quantified costs of 2-stage revision arthroplasty at the time of initial spacer placement through the 90-day, 1-year, and 2-year periods.

The present study demonstrates median total costs at 1 and 2 years after spacer placement to be $39,678 (mean = $50,304) and $44,518 (mean = $58,354), respectively. This is comparatively less than what is reported elsewhere in the literature for American studies. A large retrospective study by Kurtz et al. [4] using the Nationwide Inpatient Sample in 2012 identified an average cost of $30,300 (2009 USD) for the index surgical stay of a hip PJI treatment [4]. This is less than the median cost of $15,246 for the hospital stay associated with initial spacer placement identified in the present study. Importantly, the costs reported by Kurtz et al. did not include longitudinal costs, were not stratified by index procedure, and were only an estimate based on an aggregate cost-to-charge ratio. Kapadia et al. reported mean total charges of $88,623 at 1 year for 16 patients who underwent treatment for hip PJI [23]. Bozic et al. performed a similar analysis with 25 patients undergoing 2-stage revision for hip PJI and reported 1-year mean hospital costs of $96,166, with outpatient charges reaching $48,348 [24]. In comparison, the median 1-year outpatient charge in the current study was only $23,507. It is difficult to contextualize these costs related to these findings, as the previous sample sizes are smaller and the number of patients with persistent infection was not noted.

An Australian study by Peel et al. provided a cost-analysis most similar to that of this study and arrived at a base cost for hip PJI treatment of AU$30,915, translating to ∼$26,000 USD in 2021 [25]. However, their estimates were for the 26 months following a treatment with debridement, antibiotics, and implant retention (thereby avoiding revision surgeries included in our model). While this study is not directly comparable because Australia follows a single-payor health-care system, this was the most detailed analysis performed most recently compared to ours and is relevant to compare. Thus, the authors believe the reported model is a more robust characterization of costs than that of the United States health-care system. Other cost studies that have been performed are based on costs from countries with a single-payor system and are typically retrospective database studies with limited granularity and are therefore not as comparable. Despite this fact, other 1-year cost estimates range from ∼$5000 (South Korea) [21] to ∼$15,000 (United Kingdom) [26] and $19,000 (France) [27].

Although 2-stage revision arthroplasty is currently the gold standard for treating chronic PJI, the rate of infection clearance ranges from 70% to 90% [[7], [8], [9], [10], [11], [12]]. Therefore, there is a subset of patients who fail to clear their infection either after the resection or reimplantation stage. These patients may require additional debridement surgeries, spacer exchanges, or other infection-related revision procedures [9,17]. Such complications significantly alter the course of treatment and have important implications for the cost of care, as our study found significant differences in cost between the final outcome groups at all time points considered (90-day, 1-year, and 2-year total, 1-year and 2-year ED visits), except for initial spacer placement. Unsurprisingly, patients who underwent successful reimplantation without the need for further surgery had total costs that were less than half of the costs of patients who underwent reimplantation with subsequent revision procedures ($38,865 [$29,144-49,471] vs $79,223 [$53,442-100,152], *P* = .007). Patients who underwent a Girdlestone resection arthroplasty after 2-stage reimplantation had higher total costs at 1 year than the reimplantation group and had higher costs attributable to ED visits through 2 years of follow-up. This may highlight the need for improved discharge planning and postoperative rehabilitation as this procedure substantially impacts the quality of life and ability to carry out activities of daily living.

Many studies that report on costs for 2-stage revision arthroplasty do not include cost data that account for additional treatment for persistent infection and subsequent variation in final outcome. However, Kurtz et al. [18] did note that out of the patients in their cohort with at least 12 months of follow-up, 6% required additional procedures before completing second-stage reimplantation, including additional spacer placements and debridement procedures [18]. In addition, they reported cost breakdowns by stage and interstage, finding that for patients with 2-year follow-up, the average cost accrued between stages 1 and 2 for patients who required treatment was $35,475 [18]. The average cost accrued for patients who required treatment after stage 2 was $44,428 [18]. The present study found that for patients who required additional surgeries after stage 2 reimplantation, the median cost at 2 years was $79,223. While this estimate is much higher than what Kurtz et al. reported, their cost analysis did not include the costs of surgeons, anesthesiologists, prescriptions, physical therapy, nursing home, or home care, and they used hospital-based cost-to-charge ratios rather than direct costs. This makes comparison difficult because our study utilized direct costs and included the costs of relevant ancillary services.

There are several limitations to this study. Retrospective studies are subject to several biases that limit generalizability, including misclassification and recall bias. In addition, all cost data were derived from a single institution in the Southeast, and there has been reported variability in the cost of PJI revisions based on geography. However, the additional data that could be gathered from this institutional cohort provide a much more detailed picture of an episode of care for the treatment of hip PJI. The current cost data only include reported direct costs and not charges to payors, but in this case, this cost data may provide a more accurate picture of health-care resource utilization and overall economic burden. The cohort of the study was impacted significantly by incomplete follow-up and cost data, decreasing the sample size by almost half. This may be due in part to the nature of being treated at a tertiary referral center where patients often travel far for the surgical procedure but seek follow-up care closer to home.

## Conclusion

This study identified the median total 1- and 2-year costs for patients undergoing 2-stage revision arthroplasty for hip PJI to be $39,678 and $44,518, respectively. Furthermore, findings demonstrated that undergoing additional treatment for persistent infection and/or other complications can have important implications for the total cost of the episode of care. When stratified by the final outcome group (reimplantation, reimplantation with revision, spacer, and Girdlestone/resection arthroplasty), there were significant differences in all cost groupings (total, ED visits, total postoperative), except for initial spacer placement. Overall, the treatment of hip PJI with 2-stage revision and antibiotic spacer can be associated with substantial costs, especially when complicated by additional treatments and procedures.

## Conflicts of interest

Dr. Seyler receives royalties from Total Joint Orthopedics, Inc.; Pattern Health, and Restor3D; is a paid consultant for Smith & Nephew, Total Joint Orthopedics, Inc.; and Heraeus; receives research support from Next Science and Zimmer; receives publishing royalties or financial or material support from Lippincott Williams and Wilkins; and is a member of the American Association of Hip and Knee Surgeons and Musculoskeletal Infection Society. All other authors declare no potential conflicts of interest.

For full disclosure statements refer to https://doi.org/10.1016/j.artd.2022.10.011.

## Appendix A Supplementary Data

Conflict of Interest Statement for Charalambous

Conflict of Interest Statement for Cochrane

Conflict of Interest Statement for Kim

Conflict of Interest Statement for Seyler

Conflict of Interest Statement for Wixted

Conflict of Interest Statement for Belay

Conflict of Interest Statement for Joseph
