# Supplementary material for: Direct Costs Vary by Outcome in Two-Stage Revision Arthroplasty for the Treatment of Hip Periprosthetic Joint Infection
Source: Arthroplast Today. 2022 Nov 28;19:101061. doi: 10.1016/j.artd.2022.10.011 (PMC9713268; doi:10.1016/j.artd.2022.10.011)
Supplement: Conflict of Interest Statement for Wixted [file mmc5.docx]

# INDIVIDUAL CONFLICT OF INTEREST STATEMENT

***American Association of Hip and Knee Surgeons***

(Adopted from the American Academy of Orthopaedic Surgeons disclosure statement)

The following form **must be filled out completely and submitted by each author (example, 6 authors, 6 forms).**

**All items require a response. If there is no relevant disclosure for a given item, enter "*None*.”**

**Direct Costs Vary by Outcome in Two-Stage Revision Arthroplasty for the Treatment of Hip Periprosthetic Joint Infection**

**Manuscript Title**

1. Royalties from a company or supplier: NONE

2. Speakers bureau/paid presentations for a company: NONE

3A. Paid employee for a company or supplier: NONE

3B. Paid consultant for a company: NONE

3C. Unpaid consultants for a company or supplier: NONE

4. Stock or stock options in a company or supplier: NONE

5. Research support from a company or supplier as a Principal Investigator: NONE

6. Other financial or material support from a company or supplier: NONE

7. Royalties, financial or material support from publishers: NONE

8. Medical/Orthopaedic publications editorial/governing board: NONE

9. Board member/committee appointments for a society: NONE

Colleen Wixted Colleen Wixted June 18, 2022

Author Name (Print or Type) Author Signature Date
